# Recent Advances in Metal-Organic Frameworks for Electromagnetic Wave Absorption

**DOI:** 10.34133/research.0876

**Published:** 2025-09-08

**Authors:** Xue Zhang, Gongming Xin, Na Wu, Fei Pan, Jiurong Liu, Zhihui Zeng

**Affiliations:** ^1^ Key Laboratory for Liquid–Solid Structural Evolution and Processing of Materials (Ministry of Education), School of Materials Science and Engineering, Shandong University, Jinan 250061, China.; ^2^ School of Nuclear Science, Energy and Power Engineering, Shandong University, Jinan 250061, China.; ^3^ School of Chemistry and Chemical Engineering, Shandong University, Jinan 250100, China.; ^4^ Department of Chemistry, University of Basel, Basel 4058, Switzerland.; ^5^ Key Laboratory for Metamaterial and Electromagnetic Manipulation Technology of Shandong Province, Shandong University, Jinan 250061, China.; ^6^ State Key Laboratory of Coatings for Advanced Equipment, Shandong University, Jinan 250061, China.; ^7^ Shenzhen Research Institute of Shandong University, Shenzhen 518057, China.

## Abstract

With the rapid advancement of communication technologies, issues of electromagnetic pollution and electromagnetic compatibility have become increasingly severe, heightening the demand for high-performance electromagnetic wave absorption materials. Metal-organic frameworks (MOFs) have flourished in this field owing to their chemical tunability, high porosity, tailored topological structures, and functionality. MOF-derived composites exhibit diverse loss mechanisms and heterogeneous structures, achieving lightweight, broadband, and highly efficient absorption. Notably, recent developments in conductive MOFs (cMOFs) have positioned pristine MOFs as promising intrinsic absorbers. Accordingly, this review comprehensively classifies and summarizes recent progresses in MOF derivatives and cMOF-based absorbers, with a focus on 3 critical aspects: design strategies (compositional and structural engineering), absorption performance (reflection loss and bandwidth), and loss mechanisms (dielectric and magnetic loss). Finally, perspectives on future development directions for MOF-based absorption materials are proposed. This review provides methodological guidelines for constructing high-performance MOF-based absorption materials in the future, while highlighting persisting challenges in their development. Ultimately, it charts a course toward designing and fabricating lightweight, broadband, and high-efficiency MOF absorption materials with structural–functional integration.

## Introduction

Electromagnetic waves (EMWs), serving as a means of energy transfer and a carrier for information transmission, play a crucial role in various fields such as wireless communications and electronic devices [1–3]. In recent years, the rapid development of 5G technology, centered around EMWs, has further enhanced information propagation efficiency [4–6]. This progress has brought significant convenience to people’s production and daily lives, exerting a profound impact on economic and social development. However, the advancement of EMW-related technologies is a double-edged sword. Their flourishing development has led to a substantial increase in power density within human society, resulting in increasingly severe problems of electromagnetic interference and radiation [7–9]. Thus, developing EMW absorption materials to address these challenges is highly desired.

**Fig. 1.**
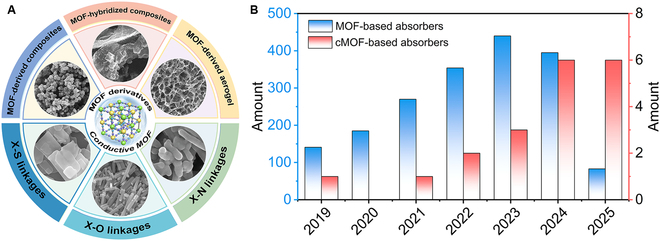
The current development status of MOF-based absorption materials. (A) MOF-based EMW absorption materials. (B) Number of published items on MOF-based materials and cMOF-based materials as EMW absorption materials from 2019 to 2025. (A) Reproduced with permission from Refs. [39,48,53,63,68,75].

Metal-organic frameworks (MOFs) are constructed through the coordination of metal ions and organic linkers, which can provide a platform for precisely tuning composition and structure, characterized by high specific surface area, porosity, tunable topology, and chemical adjustability. These properties have attracted significant interest in the field of EMW absorption materials [10,11]. Compared to traditional wave absorption materials such as ferrites, carbonyl iron, and magnetic metals, MOFs exhibit superior performance in reducing material density, enhancing absorption intensity, and broadening absorption bandwidth. Specifically, using MOF as the precursor, through thermal decomposition, derivatives with unique network structures and highly dispersed components can be constructed. These derivatives possess abundant defect structures and heterostructures, facilitating the synergistic effect of multiple loss mechanisms. Currently, MOF-based material systems have been developed as core components in this field (Fig. 1). These systems demonstrate promising application prospects and have contributed substantially to the advancement of wave absorption materials.

Typical MOF materials primarily include the IRMOF series (isoreticular metal-organic frameworks) [12], the MIL series (Materials of Institute Lavoisier framework) [13], the CPL series (coordination pillared layer materials) [14], the ZIF series (zeolitic imidazolate frameworks) [15], and the UiO series (Zr-based MOFs with tetravalent nodes) [16]. Beyond these extensively studied classical MOFs, additional series such as PCN (porous coordination network), NU-1000 (Northwestern University), and HKUST-1 (Hong Kong University of Science and Technology) are also significant [17,18]. Traditional MOFs achieve coordination bonding through metal ions linked to oxygen or nitrogen atoms with low redox activity. This results in substantial charge transfer barriers and insufficient free charge carriers, exhibiting electrical conductivities below 10^−10^ S·cm^−1^ and behaving as insulators [19]. Consequently, their EMW dissipation capability is severely limited. To enhance loss ability, the derivatives obtained after MOF carbonization/sulfurization/phosphorization treatment are usually used as EMW absorption materials. MOF derivatives can be further combined with highly conductive materials such as reduced graphene oxide (rGO), carbon nanotubes (CNTs), and Ti_3_C_2_T*_x_* (MXene) to enhance the loss capacity of the material [20–22]. This strategy enables the development of high-efficiency EMW absorption materials. Based on this, structure–function integrated EMW absorption materials are further constructed to meet the urgent demands of practical applications. In recent years, π–d conjugated MOFs, constructed by metal ions and conjugated organic ligands (where hybridization occurs between the π-orbitals of ligands and d-orbitals of metal ions), have achieved enhanced intrinsic conductivity of MOFs [23,24]. Consequently, these conductive MOFs (cMOFs) demonstrate substantial potential in applications including batteries [25], supercapacitors [26], chemical sensors [27], and electrocatalysis [28]. In particular, the development of cMOFs has also opened up new avenues for the field of EMW absorption. Notably, cMOF-based EMW absorption materials have recently emerged as a promising research focus.

Consequently, this review comprehensively summarizes the current research landscape of EMW wave absorption materials by classifying them into 2 primary categories as shown in Fig. 1A: (a) MOF-derived EMW wave absorption materials subcategorized as MOF-derived composites, MOF-hybridized composites, and MOF-derived aerogels, and (b) cMOF EMW absorption materials classified according to ligand coordination atoms (S/O/N-coordinated systems). We systematically summarize their design and synthesis methods, EMW absorption performance, and loss mechanisms, concluding with perspectives on future development to provide theoretical guidance and insights for researchers studying MOF-based absorption materials.

## EMW Absorption Theory

The 2 critical factors determining EMW absorption performance are the impedance matching characteristics and the attenuation capability. Impedance matching characteristics determine the extent to which EMWs can enter the absorber. Attenuation capability determines the degree to which the EMWs entering the material are dissipated. The loss mechanisms primarily arise from dielectric loss and magnetic loss. Dielectric loss mainly includes conductive loss and polarization loss. Within the gigahertz (GHz) frequency range, polarization loss is primarily attributed to interfacial polarization–relaxation loss and dipole polarization–relaxation loss. Magnetic loss mainly includes natural resonance, exchange resonance, and eddy current loss [29].

## MOF-Derived EMW Absorption Materials

Owing to the crystalline nature of MOFs formed through coordination bonding between metal ions and organic ligands, MOFs can serve as precursors to derive EMW wave absorption materials with multiple loss mechanisms. By further combining the MOF derivatives with highly conductive materials, a heterostructure can be effectively constructed, impedance matching can be optimized, and the loss capacity can be enhanced, thereby obtaining highly efficient EMW absorption materials.

### MOF-derived composites

In 2015, Qiang et al. [30] reported Prussian blue-derived Fe/C by controlled high-temperature pyrolysis, which achieved high-efficiency EMW absorption performance with a minimum RL value of −22.6 dB at 15.0 GHz with a thickness of 2 mm at the filler loading of 40 wt%. Meanwhile, Lu et al. and Zhang et al. [31,32] prepared Co/C and FeCo/C nanocomposites for EMW wave absorption by carbonizing the ZIF-67. Since then, through thermal treatment of carbonization, sulfurization, or phosphorization on MOFs, the organic frameworks undergo carbonization while metal ion centers are reduced to corresponding metal nanoparticles or transformed into metal oxides/sulfides/phosphides, thereby forming carbon-based composites with uniformly dispersed metal/oxide/sulfide/phosphide phases, from which 1-component, 2-component, 3-component, and higher-component composite absorbing materials can be obtained [33–37].

**Fig. 2.**
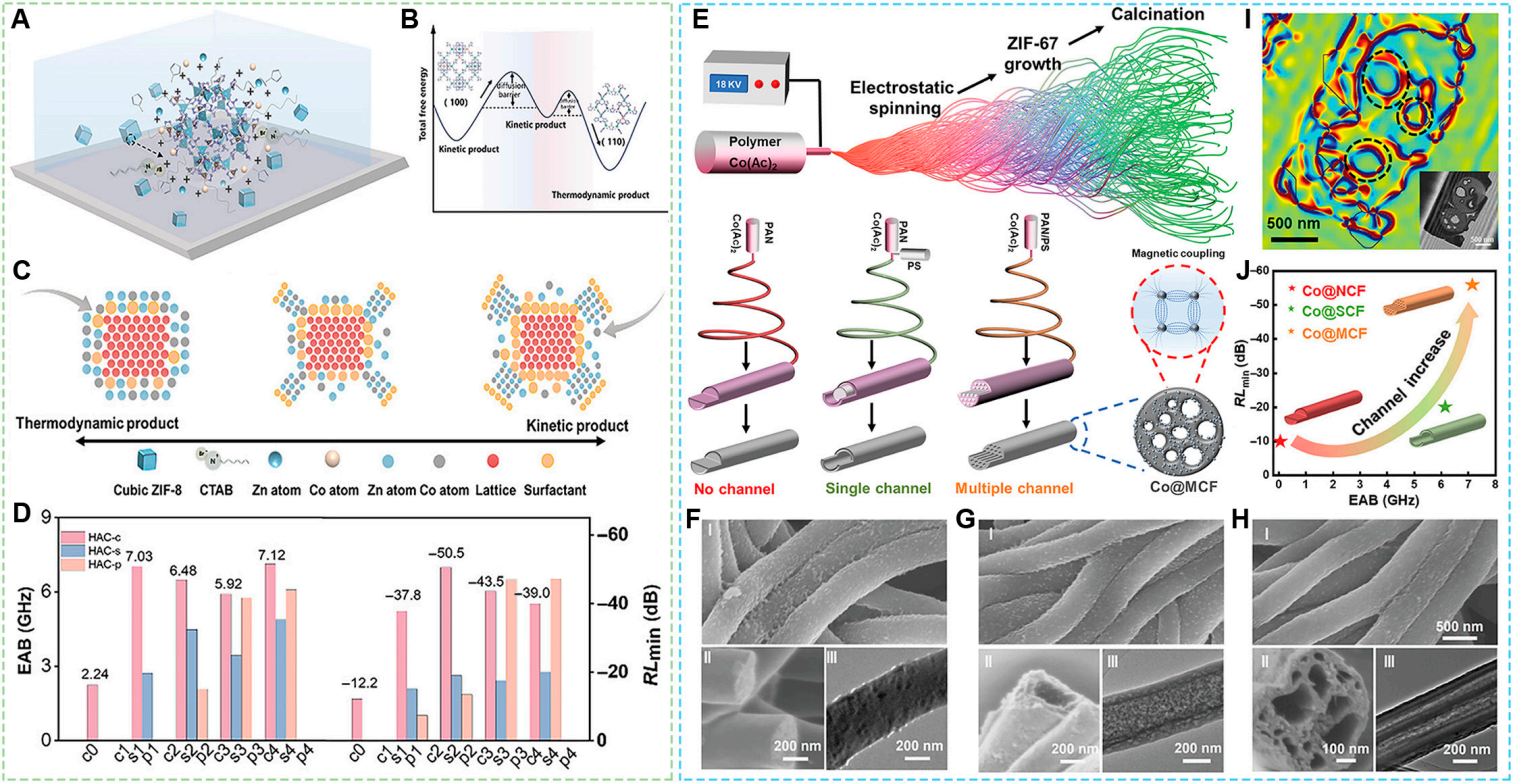
EMW absorption materials derived from ZnCo-ZIF and ZIF-67. (A) Schematic illustration of the atomic deposit on the cubic-ZIF-8 crystal seed surface. (B) A schematic illustration depicting the thermodynamic and kinetic products in terms of total free energy, as well as the diffusion energy barrier associated with the transformation from the kinetic product to the thermodynamic product. (C) Schematic illustration of the growth mechanism of oriented MOFs. (D) The EAB and minimum RL comparison between Co/ZnO/Co_3_ZnC with different crystal orientations. (E) Schematic illustration of the fabrication process and detailed mode in Co@muti-channel carbon fibers. (F to H) Scanning electron microscopy (SEM) and transmission electron microscopy (TEM) images of Co@NCF, Co@SCF, and Co@MCF. (I) Off-axis electron holograms image of the cross-section of Co@MCF. (J) The comparison of EMW absorption performance before and after channel regulation in Co@carbon fibers. (A to D) Reproduced with permission from Ref. [40]. Copyright 2025, Wiley-VCH. (E to H) Reproduced with permission from Ref. [44]. Copyright 2025, Wiley-VCH.

Recent years have witnessed intensified research focus on elucidating the impact mechanisms of microstructure regulation on EMW absorption performance. For instance, Wang et al. enabled the morphological evolution of Co element from single atoms to atomic clusters and ultimately nanoparticles through precise control of metal atoms and orbital hybridization in ZnCo-ZIF, which effectively modulates polarization loss [38]. The Co embedded nitrogen-doped carbon matrix harvested an EMW absorption performance with a minimum RL of −54.4 dB and an EAB of 8.4 GHz. In addition, Zhang et al. [39] also reported that MOF-derived Ni single-metal atoms dispersed hierarchically porous carbon nanoflowers. The composites exhibited a minimum RL of −53.2 dB and an EAB of 5.0 GHz at the filler loading of 10 wt%. Cui et al. [40] studied the interdependency between crystal plane orientation and intrinsic electromagnetic properties in ZnCo-ZIF-derived Co/ZnO/Co_3_ZnC heteroatomic metal nanoclusters as shown in Fig. 2A to D. The results showed that the Co_3_ZnC played a dominant role in EMW attenuation, improving conductive loss along the (110) plane orientation, while enhancing polarization losses along the (200) plane orientation. As a result, the nanocomposites could obtain the EAB of 7.1 GHz with a thickness of 2.3 mm. Yan et al. [41] reported a CeNiCo-MOF-derived Ce/NiCo@C composite with a radial dielectric gradient structure, in which the dielectric constant and electron migration polarization could be modulated and promoted. As a result, the composites could achieve a minimum RL of −67.2 dB at a thickness of 1.94 mm and an EAB of 7.12 GHz. Huang et al. [42] prepared FeZn-ZIF-derived ZnFe_2_O_4_-ZnO-Fe@C microspheres with custom-built heterogeneous interfaces. The microspheres exhibited a minimum RL of −66.5 dB at a thickness of 2.0 mm. Liu et al. [43] prepared MIL-88A@PVP@ZIF-67-derived hierarchical Fe-Co@TiO_2_ with incoherent heterointerfaces and gradient magnetic domains. The Fe-Co@TiO_2_ absorbents exhibited a minimum RL of −51.5 dB at a thickness of 2.5 mm, and a maximum EAB of 8.6 GHz, covering the whole Ku band and most of the X band. Moreover, as shown in Fig. 2E to H, Wang et al. [44] prepared Co uniformly embedded carbon framework with a 1D multichannel structure using a combination of electrospinning, in situ growth of ZIF-67, and a subsequent high-temperature annealing for EMW absorption. This work demonstrated that the magnetic components were uniformly distributed in the carbon skeleton, promoting the construction of a magnetic coupling network within and between fibers, effectively enhancing the magnetic loss effect. Meanwhile, the multichannel structure formed a large number of heterogeneous interfaces, which could effectively enhance the polarization loss. As a result, the composites delineated a minimum RL of −57.65 dB and a maximum EAB of 7.28 GHz at a thickness of 1.9 mm.

### MOF-hybridized composites

In addition to the aforementioned EMW absorption derived from MOFs, researchers often combine MOF derivatives with highly conductive materials such as graphene, CNTs, and MXene, which leverage exceptional specific surface area, superior electrical conductivity, and abundant functional groups to amplify conductive dissipation [9,45,46]. Meanwhile, it is conducive to the further construction of multiple heterogeneous interfaces to improve polarization loss, thereby optimizing the EMW absorption performance. Therefore, this strategy has broad application prospects in the field of EMW absorption.

**Fig. 3.**
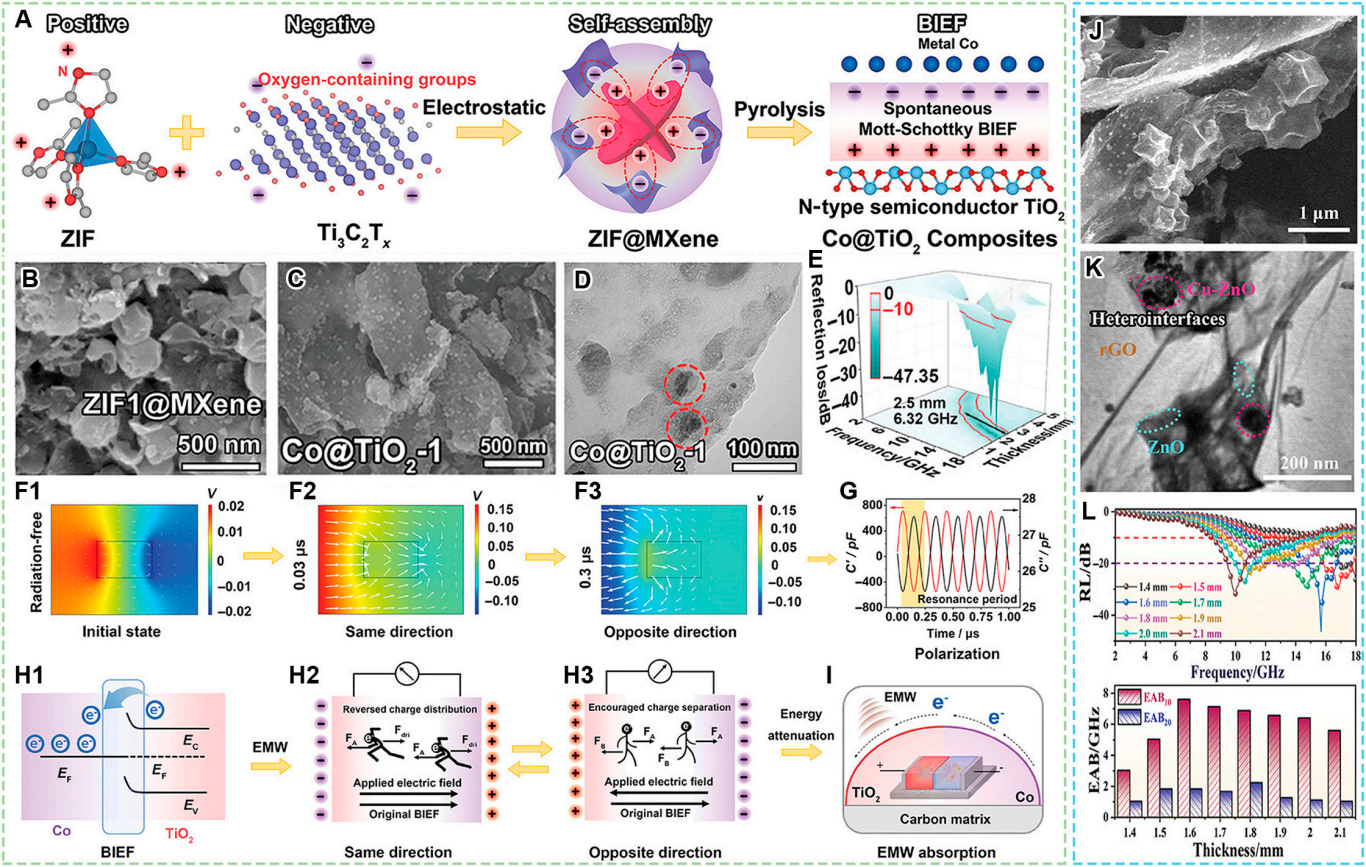
EMW absorption materials derived from MOF hybridized with MXene and rGO composites. (A) Electrostatic self-assembly of ZIF@MXene and generation of the built-in electric field (BIEF) in Co@TiO_2_ nanocomposites. (B) SEM images of ZIF@MXene. (C and D) SEM and TEM images of Co@TiO_2_. (F) Spatial charge distribution in BIEF under D_10GHz_ radiation. (G) Maxwell capacitance of BIEF under D_10GHz_ radiation. (H1) Schematic of band alignment and electron redistribution in the Co–TiO_2_ heterointerface. Mechanism underlying interfacial charge transport in scenarios where the applied electric field is (H2) oriented along or (H3) opposite to the original BIEF. (I) Schematic illustrating the comprehensive EMW absorption mechanism of the Co@TiO_2_ nanocomposite. (J and K) SEM and TEM images of Cu/ZnO/rGO composites. (L) 2D RL curves (top) and corresponding EAB_10_ and EAB_20_ of Cu/ZnO/rGO composites at different thickness (<2.1 mm). (A to I) Reproduced with permission from Ref. [47]. Copyright 2024, Wiley-VCH. (J to L) Reproduced with permission from Ref. [48]. Copyright 2025, Wiley-VCH.

As shown in Fig. 3A to I, Gao et al. [47] reported ZIF@MXene-derived Co@TiO_2_ with abundant heterointerfaces. This work elucidated that the abundant Mott–Schottky heterointerfaces exhibited a robust built-in electric field (BIEF) effect, which proficiently altered charge separation, facilitated electron migration, and ultimately enhanced polarization relaxation loss. As a result, the composites could achieve a minimum RL of −47.35 dB and a maximum EAB of 6.32 GHz. Zhang et al. [48] also prepared CuZn-ZIF-derived Cu/ZnO/rGO composite with Mott-Schottky heterojunctions through the construction of BIEF (Fig. 3J to L). This work revealed that there always coexisted both enhanced charge separation and reversed charge distribution in this double BIEF, boosting the interface polarization. The composites could achieve a minimum RL of −46.29 dB and a maximum EAB of 7.6 GHz at a thickness of 1.6 mm. Zhai et al. [49] reported V_2_O_3_/VO/C@Ti_3_C_2_T*_x_*/TiO_2_ composites through carbonization of modified MIL-88B(V) with MXene, which also achieved the regulation of electron transport properties by building the BIEF. The composites could achieve a minimum RL of −50.10 dB and a maximum EAB of 6.16 GHz at the filler loading of 20 wt%.

### MOF-derived aerogels

MOF derivatives have achieved significant breakthroughs in regulating electromagnetic parameters and optimizing loss mechanisms. However, in practical applications, traditional EMW absorption agents need to be coated with adhesives or filled into the matrix, which leads to an increase in material thickness and weight rise. The interface compatibility issue is prone to cause coating detachment or performance degradation. As a result, it is difficult to meet the urgent demands for lightweight and integrated structure–function in actual applications. Therefore, researchers have conducted studies on integrated structural–functional EMW absorption materials [50–52]. By synergistically combining the EMW absorption agent and the structural matrix at the mesoscopic scale, these materials enabled simultaneous mechanical robustness and electromagnetic dissipation.

**Fig. 4.**
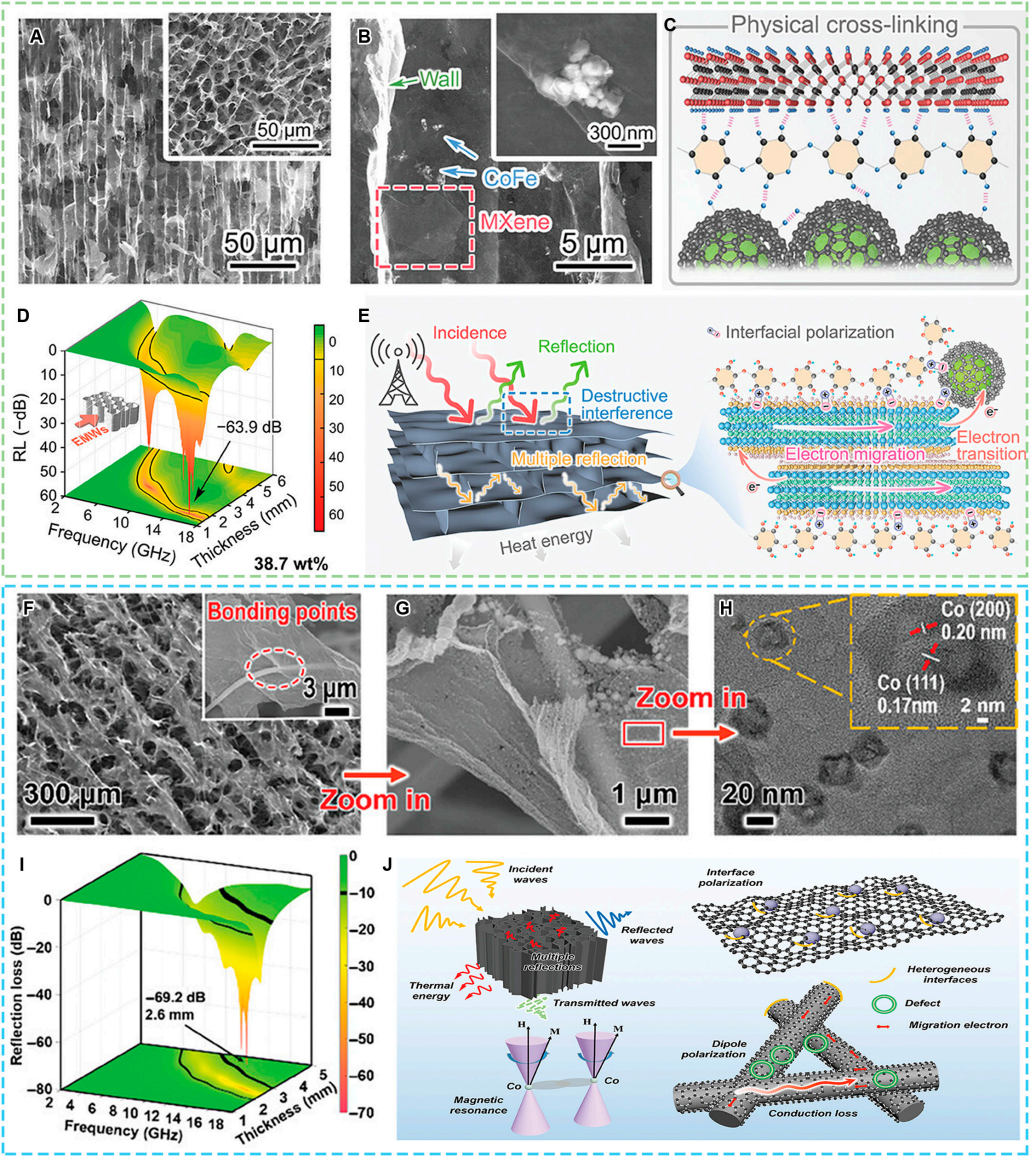
MOF-derived aerogels as EMW absorption materials. (A) SEM images of the biomimetic ordered CoFe/C/MXene/CNF aerogel and transverse (inset) section. (B) SEM images of cell walls showing the distribution and binding state between magnetic nanoparticles and MXene. (C) The cross-linking mechanism. (D) 3D RL curves of the CoFe/C/MXene/CNF aerogel. (E) Schematic illustrations of EMW absorption mechanisms for the CoFe/C/MXene/CNF aerogel. (F to H) SEM and TEM images of the SiO_2_/rGO/Co aerogel. (I) 3D RL representation of the SiO_2_/rGO/Co aerogel. (J) Schematic illustration of the EMW attenuation mechanism for the SiO_2_/rGO/Co aerogel. (A to E) Reproduced with permission from Ref. [53]. Copyright 2024, Wiley-VCH. (F to J) Reproduced with permission from Ref. [55]. Copyright 2024, Wiley-VCH.

As shown in Fig. 4A to E, Qiao et al. [53] constructed a CoFe-Prussian blue analogue (PBA)-derived CoFe/C/MXene/cellulose nanofibrils (CNFs) aerogel with a density as low as 12 mg cm^−3^. Benefiting from the physical and chemical dual cross-linking, the aerogel acquired a mechanical strength of up to 1,311%. The aerogel performed a minimum RL of −63.9 dB at a thickness of 1.7 mm and a maximum EAB of 5.2 GHz at a thickness of 1.9 mm. Besides, by carbonizing the CoFe-PBA-anchored CNFs, Qiao et al. [54] also prepared porous carbon aerogels with CoFe@C magnetic nano-capsules. The aerogel could achieve a minimum RL of −70.8 dB and a maximum EAB of 6.0 GHz. Li et al. [55] prepared magnetically conductive ceramic nanofibrous aerogel composed of silicon dioxide nanofibers, graphene, and ZIF-67 derivatives (Fig. 4F to J). The SiO_2_/rGO/Co aerogel with a density of 4.3 mg cm^−3^ depicted a minimum RL of −69.2 dB at a thickness of 2.6 mm and a maximum EAB of 7.51 GHz at 3.2 mm. The aerogels exhibited high thermal stability and insulation properties, making them highly applicable to the environment. Besides, Lin et al. [56] constructed MIL-101-derived magnetic γ-Fe_2_O_3_@C/graphene aerogels. Due to the synergy between the dielectric and magnetic components, the aerogel achieved a minimum RL of −60.5 dB and a broad EAB of 7.76 GHz.

### Summary

Current research on MOF-derived EMW absorption materials reveals that the intrinsic porosity, chemical tunability, and low density of pristine MOFs have propelled extensive investigations into their EMW absorption capabilities. Substantial evidence confirms that MOF derivatives achieve exceptional EMW absorption performance with enhanced loss capabilities, improved absorption intensity, and broadened bandwidth. By constructing the EMW absorption aerogel, the environmental applicability of MOF-derivative EMW absorption materials has been effectively improved, which is conducive to the practical application of EMW absorption materials. Nevertheless, the indispensable high-temperature treatment process inevitably destroys the original MOF microstructure, resulting in underutilization of their unique topological structure and obscuring the microstructure–performance correlation mechanisms.

More importantly, the research on MOF-derived EMW absorption materials is still confined to the traditional approach of achieving synergy of multiple loss mechanisms through material composites, making it difficult to achieve a breakthrough in the development of new EMW absorption materials. Consequently, focusing on exploiting the inherent electromagnetic dissipation of pristine MOF materials rather than their derivatives enables full leverage of their topological and crystal microstructure advantages. This approach can provide novel insights into structure–performance relationships and effectively advances the development of next-generation EMW absorption materials.

## cMOF-Based EMW Absorption Materials

cMOFs are an emerging class of layered frameworks featuring in-plane π–d conjugation and out-of-plane π–π stacking, exhibiting distinctive properties such as designable charge transport and tunable bandgaps. cMOFs require metal ions with high-energy electrons or holes and organic ligands bearing stable radicals or redox-active moieties to provide unpaired electrons, enabling efficient charge transfer between metallic centers and organic linkers [20]. Charge transport in cMOFs occurs via 2 pathways: hopping transport and band transport. There are 2 approaches toward providing charge transport pathways in cMOFs: a through-bond approach and a through space approach [57–59]. The π-conjugated ligands in cMOFs contain abundant unsaturated bonds and functional groups that can also enhance the loss capacity of cMOFs. Consequently, cMOFs can be a promising candidate for EMW absorption.

### cMOFs constructed by Cu-S linkages

In 2019, Wu et al. [60] reported a conductive Cu-S network (ligand is 4-hydroxybenzenethiol) for EMW absorption. Under the filler loading of 40 wt%, the minimum RL value of Cu-S can reach −56 dB at 7.76 GHz and the EAB can reach up to 6.16 GHz. The high-efficiency EMW absorption performance can be mainly attributed to the conductive loss of the Cu-S network. Miao et al. [61] reported semiconductive MOF (CuHT, HT = 4-hydroxthiophenol) without any pyrolysis at high temperature for EMW absorption. Attributed to the intrinsic resistance loss by Cu_2_S layers, the minimum RL could achieve −50.9 dB and EAB could reach 4.2 GHz (7.7 to 10.9 GHz) at the filler loading of 50 wt%. Subsequently, Miao et al. [62] again reported semiconductive Cu-S-MOF (ligands are 6,6′-dithiodinicotinic acid and pyrazine) as EMW absorption materials. The EAB of the Cu-S-MOF can obtain a maximum EAB of 6.72 GHz (9.68 to 16.4 GHz) at a thickness of 1.5 mm and a minimum RL value of −52.8 dB at 1.69 mm.

**Fig. 5.**
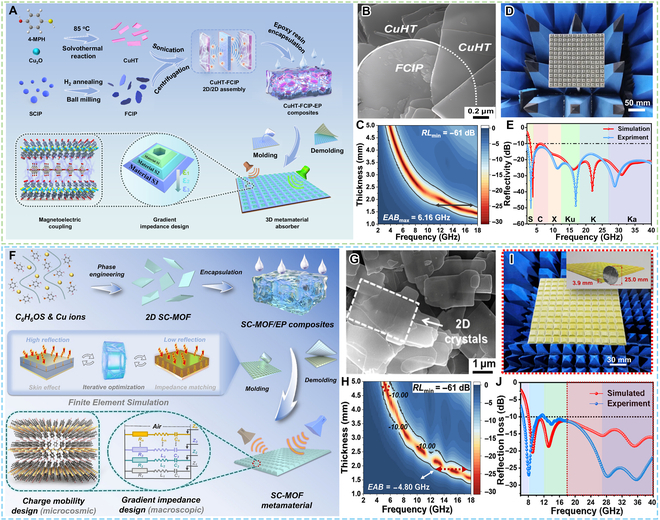
cMOFs constructed by Cu-S linkages. (A) Schematic illustration of the preparation of CuHT-FCIP-EP metamaterial absorber. (B) SEM images of CuHT-FCIP composites. (C) 2D contour diagrams of RL value versus frequency at different thicknesses for CuHT-FCIP-EP composites. (D) Photo of the CuHT-FCIP-EP metamaterial. (E) Comparison of experimental reflectivity and simulated reflectivity of ultra-broadband metamaterial absorber. (F) Schematics of the fabrication of CuHT metamaterial absorbers. (G) SEM image of CuHT. (H) 2D RL contour maps of CuHT/EP composites. (I) Photo of the CuHT/EP metamaterial. (J) Comparison of experimental reflectivity and simulated reflectivity of the CuHT/EP metamaterial. (A to E) Reproduced with permission from Ref. [63]. Copyright 2024, Springer Nature. (F to J) Reproduced with permission from Ref. [64]. Copyright 2025, Wiley-VCH.

As shown in Fig. 5A to E, Qu et al. [63] prepared sandwich-like CuHT-FCIP (HT = 4-mercaptophenol, FCIP = flake–layered carbonyl iron powders) 2D/2D assembly with abundant heterojunctions and strong magneto-electric coupling networks. This work demonstrated that the crystal/crystal heterojunction of CuHT-FCIP facilitated the extraction of electrons from the CuHT surface by the C atoms belonging to FCIP, which could promote the generation of numerous interlayer charge-transfer pathways and thus lead to a strong magnetoelectric coupling loss. The CuHT-FCIP was encapsulated in epoxy resin to fabricate the CuHT-FCIP-epoxy resin (EP) composites and metamaterial. The CuHT-FCIP-EP composites could achieve a maximum EAB of 6.16 GHz and a minimum RL of −61 dB at 5.2 GHz. The metamaterial harvested an ultra-broad bandwidth spanning 2 to 40 GHz. Moreover, Qu et al. [64] (Fig. 5F to J) further reported CuHT dispersed within EP to construct trapezoidal structures. The CuHT/EP composite could achieve a minimum RL of −61dB and a maximum EAB of 4.8 GHz. Benefiting from the scattering topological design further enhancing causality efficiency and robustness, the CuHT/EP metamaterial could achieve an ultra-wide EAB of 33.4 GHz at a thickness of 3.9 mm. Besides, Wang et al. [65] reported that CuHT arranged a CNT composite with a 1D/2D heterostructure through a one-step in situ polymerization method. The composite could obtain a maximum EAB of 6.36 GHz and a minimum RL value of −59.24 dB at 14.04 GHz with the filler loading of 40 wt%.

### cMOFs constructed by X-O (X = Zn, Ni, Co, Cu, Fe) linkages

**Fig. 6.**
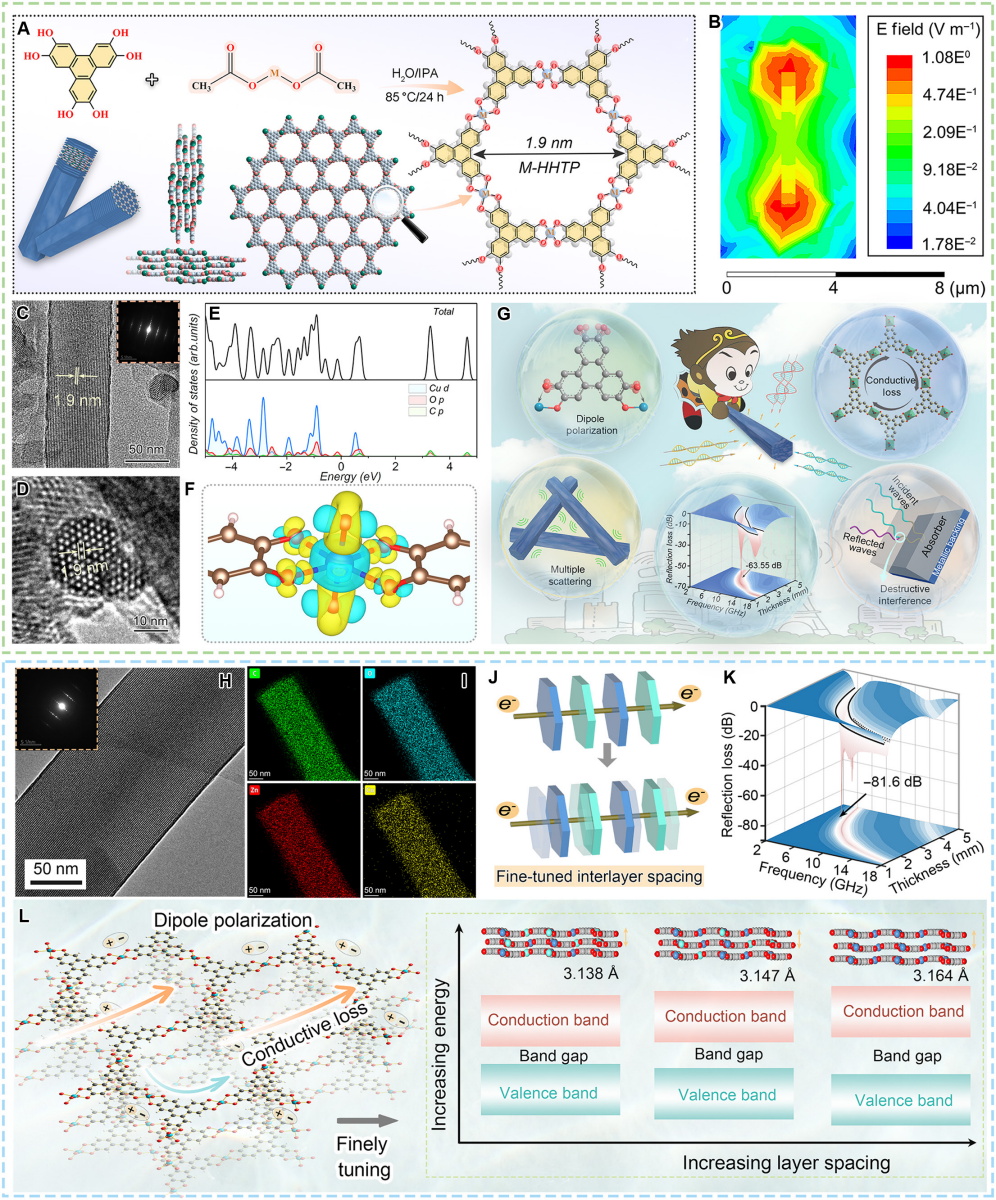
cMOFs constructed by X-O (X = Zn, Ni, Co, Cu, Fe) linkages. (A) Synthetic illustration and crystal structures of the M-HHTP (M = Zn, Cu, Co, or Ni). (B) Electric field distribution in the Zn-HHTP rod. (C and D) TEM images and selected-area electron diffractions of Zn-HHTP. (E) The density of states of the Cu-HHTP. (F) The charge density difference between the Cu-HHTP, HHTP, and Cu. (G) The schematic illustration of the EMW absorption mechanisms for the cMOFs. (H and I) The aberration-corrected HAADF-STEM images and corresponding elemental mappings of Zn3Cu1-HHTP. (J) The schematic diagram of interlayer charge transport in cMOFs. (K) The 3D RL values of Zn3Cu1-HHTP. (L) The schematic illustration of the EMW absorption mechanisms for the bimetallic cMOFs. (A to G) Reproduced with permission from Ref. [68]. Copyright 2023, American Chemical Society. (H to L) Reproduced with permission from Ref. [69]. Copyright 2024, American Association for the Advancement of Science.

Using a mixed solvent system of water and DMF, Dong et al. [66] reported 2D cMOFs [M_3_(HHTP)_2_, M = Zn, Ni, and Co; HHTP = 2,3,6,7,10,11-hexahydroxytriphenylene] as EMW absorbers. By adjusting the metal centers, the EM parameters and EMW absorption performance of cMOF could be tuned, in which Co_3_(HHTP)_2_ exhibited the best EMW absorption with a minimum RL of −60.6 dB at 11.76 GHz and a maximum EAB of 5.45 GHz in the filler loading of 60 wt%. Similarly, Shan et al. [67] also prepared M_3_(HHTP)_2_ (M = Cu, Zn, and Ni) by the hydrothermal method in an aqueous solution system. Cu_3_(HHTP)_2_ delivered a minimum RL of −56.45 dB and a maximum EAB of 5.76 GHz at a thickness of 2.1 mm while the filler loading ratio is 40 wt%. Besides, Zhang et al. [68] also synthesized HHTP-based cMOFs via the solvothermal method using a mixed solvent of water and isopropanol (Fig. 6A to J). By adjusting metal ions of Zn, Cu, Co, or Ni, tunable dielectric properties were attained to tailor EMW absorption. In particular, this work revealed that the shape anisotropy and crystallinity have a significant effect on the attenuation ability of cMOF. As a result, Cu-HHTP depicted a minimum RL value of −63.55 dB at a thickness of 2.9 mm and an EAB of 5.2 GHz with a filler loading of 50 wt%, while Zn-HHTP showcases the absorption superiority in the S band with a minimum RL value of −62.8 dB at a thickness of 1.9 mm. The prominent EMW absorption of the M-HHTP can be attributed to the conductive and polarization losses derived from the conjugation effects and terminal groups, as well as shape anisotropy. Furthermore, as shown in Fig. 6H to L, Zhang et al. [69] synthesized bimetallic cMOF of ZnCu-HHTP for fine-tuned dielectric property and EMW absorption. This work proposed a generic fine-tuned nanotechnology involving metal ions with a different radius to regulate the interlayer spacing. In particular, with the increase of Zn, the interlayer spacing continuously decreased, resulting in enhanced dielectric property. Zn3Cu1 with the appropriate dielectric property delivered the optimal EMW absorption with a minimum RL of −81.62 dB and an EAB of 3.7 GHz at a filling ratio of 50 wt %.

Moreover, Qu et al. [70] prepared CoCu-HHTP, resulting in excellent EMW absorption performance with a maximum EAB of 6.16 GHz and a minimum RL of −61 dB. The metamaterial absorber constructed by encapsulating the CoCu-HHTP in polydimethylsiloxane demonstrates a bandwidth of 11.33 GHz. Qu et al. [71] also synthesized CoNi-CAT for EMW absorption, leveraging the precise control of interlayer spacing in SC-MOFs for electron mobility regulation and the introduction of paraffin wax for temperature-inert electromagnetic properties. Consequently, the Co_0.6_Ni_0.4_-HITP could achieve a maximum EAB of 5.52 GHz at a thickness of 2.04 mm and a minimum RL of −63.43 dB with a filling ratio of 50 wt%.

Wang et al. [72] synthesized Cu-HHTP with different nanostructures for EMW absorption. The influence of the nanostructures of the cMOFs on the dielectric and EMW absorption performance was clarified. In particular, the Cu-HHTP with a nanosheet structure and high specific area exhibited high-efficiency EMW absorption performance with a minimum RL of −51.08 dB at 7.25 GHz with a thickness of 4.4 mm and a maximum EAB of 5.73 GHz at 2.5 mm. Wu et al. [73] reported Cu-HHTP@polypyrrole (PPy) with a heterostructure that brings interfacial polarization and enhanced the EMW absorption performance. The EAB could reach 6.68 GHz (11.00 to 17.68 GHz) at a thickness of 2.4 mm, and the RL could reach −59.34 dB at a thickness of 2.7 mm. Chen et al. [74] prepared Cu-TBTT [TBTT = thieno(3,2-*b*)thiophene] with a 1D and 2D topology structure by optimizing the solvent environment for EMW absorption. Benefiting from the geometric effects induced by the 1D linear topology and the synergistic absorption mechanism involving both polarization and conduction, the CuTBTT-1D exhibited optimal EMW absorption performance with a minimum RL value of −77 dB and a maximum EAB of 6.52 GHz, with a thickness of 2.2 mm and a filler loading of 15 wt%.

### cMOFs constructed by X-N (X = Cu, Ni, Fe) linkages

**Fig. 7.**
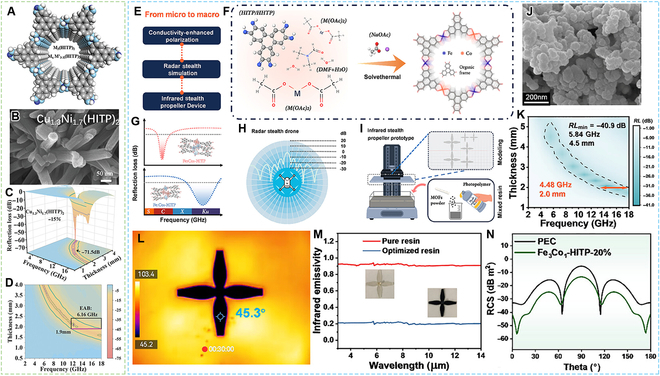
cMOFs constructed by X-N (X = Cu, Ni, Fe) linkages. (A) Top views of the molecular structure model of HITP-MOFs (M, M′ = Co, Ni, Cu). (B) SEM image of Cu_1.3_Ni_1.7_(HITP)_2_. (C and D) The 3D RL plots and corresponding 2D diagrams of Cu_1.3_Ni_1.7_(HITP)_2_. (E) Schematic diagram of the regulation strategy. (F) Bottom-up synthesis process of π-conjugated MOFs. (G) Illustration of the strongest and broadest EMA performance in Fe_3_Co_1_-HITP and Fe_1_Co_1_-HITP, respectively. (H) Radar stealth performance and (I) stereolithography 3D printing process. (J) SEM image of Fe_1_Co_3_-HITP. (K) Color-mapped RL diagram of Fe_3_Co_1_-HITP. (L) Steady-state IR thermal images of the π-conjugated MOFs-1.1 metadevice after 30 min at an ambient temperature of 100 °C. (M) Infrared emissivity of the π-conjugated MOFs-1.1 and pure resin metadevice. (N) 2D RCS plot of the Fe_3_Co_1_-HITP-coated UAV model at 4.5 mm, 5.84 GHz. (A to D) Reproduced with permission from Ref. [75]. Copyright 2023, Wiley-VCH. (E to N) Reproduced with permission from Ref. [77]. Copyright 2025, Wiley-VCH.

Chen et al. [75] reported the nitrogen-coordinated bimetallic MOFs (CuNi-HITP, HITP = 2,3,6,7,10,11-hexaimino triphenylene) as EMW absorption materials (Fig. 7A to D). Also, benefiting from the alteration of free-carrier concentration induced by the subtle difference of interlayer displacement or spacing, which originate from the atomic tuning of hetero-metal, the Cu_1.3_Ni_1.7_(HITP)_2_ delivered an ultra-high absorption performance with a minimum RL of −71.5 dB and a maximum EAB of 6.16 GHz at a filling ratio of 15 wt%. Cheng et al. [76] reported a family of Cu-based cMOFs, which is afforded by coordinating Cu with the triphenylene-X ligands (X = −NH_2_, −OH, and −SH). More specifically, due to the unique N−H bond and a more negatively conjugated C, the Cu_3_(HITP)_2_ (X = −NH_2_) could accomplish the optimal EMW absorption performance with a minimum RL of −63.03 dB at a thickness of 3.2 mm and a maximum EAB of 3.44 GHz.

Jin et al. [77] reported both FeCo-HHTP and FeCo-HITP via strengthening atom coordination, by leveraging molecular-level design, which can precisely tailor the absorption bands and effective bandwidths of MOF materials (Fig. 7E to N). Consequently, the Fe_3_Co_1_-HITP could achieve a maximum EAB of 4.48 GHz at a thickness of 2 mm and a minimum RL of −40.9 dB at a thickness of 4.5 mm with a filler loading of 20 wt%. Fe_3_Co_1_-HITP-20%-coated UAV displayed a maximum RCS reduction of −23.3 dB m^2^. Besides, the cMOF can be dispersed into electromagnetically transparent photopolymer resin to fabricate a drone propeller prototype that exhibits a remarkably low infrared emissivity of 0.205.

### Summary

Current research on cMOF-based wave absorption materials reveals that their exceptional absorption performance originated from conductive loss benefiting from electrical conductivity. The charge transport of MOFs can be modulated through the regulation of both metal ion centers and organic ligands, allowing precise control over dielectric properties and EMW absorption. Besides, modulation of the synthetic solvent system can precisely adjust MOF microstructure and crystallinity, which can enhance polarization dissipation capability. The comparison of EMW absorption performance between cMOFs is depicted in the Table 1. Notably, nitrogen-coordinated cMOFs can achieve high-efficiency EMW absorption performance at low filler loading. It is primarily because nitrogen atoms possess higher electron delocalization and stronger conjugation effects, endowing N-coordinated cMOFs with superior conductivity and enhanced loss capabilities. Thus, further improving MOF conductivity facilitates the development of lightweight absorbers. However, due to the single loss mechanism, cMOFs depicted a narrow effective bandwidth. To address this limitation, researchers have composited cMOFs with polymer binders and constructed diverse metamaterial structures via 3D printing, significantly broadening their absorption bandwidth. Consequently, for practical applications of cMOFs, greater emphasis should be placed on functional–structural integration, advancing toward lightweight, broadband absorption cMOF-based EMW absorption materials.

**Table 1.** Comparison of minimum RL and maximum EAB between cMOFs

Materials	RL (dB)	EAB (GHz)	Filler loading (wt%)	Reference
Cu-S network	−56	6.16	40	[60]
CuHT	−50.9	4.2	50	[61]
Cu-S-MOF	−52.8	6.72	100	[62]
CuHT-FCIP	−61	6.16	60	[63]
CuHT	−61	4.8	62.5	[64]
CuHT arranged carbon nanotubes (CNTs)	−59.24	6.36	40	[65]
Co_3_(HHTP)_2_	−60.6	5.45	60	[66]
Cu_3_(HHTP)_2_	−56.5	5.76	40	[67]
Cu-HHTP	−63.55	5.2	50	[68]
Zn-HHTP	−62.8	1.5	50	[68]
ZnCu-HHTP	−81.62	3.7	50	[69]
CoCu-HHTP	−61	6.16	50	[70]
CoNi-CAT	−63.43	5.52	50	[71]
Cu-HHTP	−51.08	5.73	50	[72]
Cu-HHTP@polypyrrole	−59.34	6.68	20	[73]
CuTBTT	−77	6.52	15	[74]
Cu_1.3_Ni_1.7_(HITP)_2_	−71.5	6.16	15	[75]
Cu_3_(HITP)_2_	−63.03	3.44	60	[76]
Fe_3_Co_1_-HITP	−40.9	4.48	20	[77]

## Conclusion and Perspectives

Aimed at escalating electromagnetic radiation pollution and the demand for advanced EMW wave absorption materials, the development of novel high-efficiency EMW absorption materials is urgently required. MOFs exhibit flourishing potential in this field due to their high specific surface area, compositional tunability, and topological crystal structures. The current development of MOF-based EMW absorption materials has achieved a milestone progress and MOF-based materials have achieved a highly efficient EMW absorption performance. Nevertheless, as technology continues to advance, MOF materials should also keep up with the times.

On the one hand, MOF-derived composites, endowed with superior chemical and thermal stability, could leverage heterointerfaces to synergistically optimize both impedance matching and loss mechanisms through composition and structure design, further accomplishing lightweight, broadband, highly efficient, and structural–functional integrated efficient EMW absorption materials serving as next-generation material. However, precisely controlling the MOF derivatization process to achieve tailored synthesis with optimal composition, defect engineering, and pore structure remains a substantial challenge. Furthermore, the robustness of MOF-derived absorbers under harsh operating conditions (e.g., prolonged exposure to high humidity, elevated temperatures, or corrosive environments) requires more systematic evaluation. Ensuring long-term performance stability is crucial for practical applications.

On the other hand, for cMOF materials representing an emerging and important direction, by designing metal ion centers and organic ligands, their charge transport capabilities can be enhanced to boost intrinsic conductivity. Research should elucidate how cMOF composition and structure govern charge transport characteristic, thereby revealing mechanisms determining their dielectric properties and EMW absorption performance. Understanding these structure–performance relationships enables precise modulation of conductivity and dielectric behavior, establishing a material foundation for structural–functional integrated cMOF-based EMW absorption and charting new research direction for MOF-based absorption materials. Nevertheless, it remains challenging to enhance the electrical conductivity of cMOFs while ensuring their chemical stability. Additionally, cMOF absorbers exhibit relatively singular loss mechanisms and suboptimal impedance matching, resulting in narrow absorption bandwidths that require further optimization. Furthermore, the MOF synthesis process is rather demanding and difficult to scale up for production. Future research should focus on addressing these challenges to develop more promising MOF-based EMW absorption materials.
